# Sickle-cell disease in febrile children living in a rural village of Madagascar and association with malaria and respiratory infections

**DOI:** 10.1186/s12878-016-0069-1

**Published:** 2016-12-01

**Authors:** Muriel N. Maeder, Henintsoa M. Rabezanahary, Norosoa J. Zafindraibe, Martin Raoelina Randriatiana, Tahinamandranto Rasamoelina, Andry T. Rakotoarivo, Philippe Vanhems, Jonathan Hoffmann, Thomas Bénet, Mala Rakoto Andrianarivelo, Olivat A. Rakoto-Alson

**Affiliations:** 1 Centre d’Infectiologie Charles Mérieux, Université Antananarivo, P.O. Box 4299, Antananarivo, Madagascar; 2 Fondation Médicale d’Ampasimanjeva, Manakara, Madagascar; 3 UPFR Biochimie-Centre Hospitalier Universitaire Joseph Ravoahangy Andrianavalona, Antananarivo, Madagascar; 4 Laboratoire des Pathogènes Emergents, Fondation Mérieux, Centre International de Recherche en Infectiologie (CIRI), Inserm U1111, CNRS UMR5308, ENS de Lyon, UCBL1 Lyon, France; 5 Service d’Hygiène, Epidémiologie et Prévention, Hôpital Edouard Herriot, Hospices Civils de Lyon, Lyon, France; 6 UPFR Hématologie-Centre Hospitalier Universitaire Joseph Ravoahangy Andrianavalona & Département de Microbiologie, Faculté de Médecine, Antananarivo, Madagascar

**Keywords:** Sickle-cell disease, Fever, Malaria, Respiratory Infections, Children, Madagascar

## Abstract

**Background:**

In Madagascar, the last study on sickle cell disease (SCD) was done in the early 1980s. The country is known as endemic for malaria and respiratory infections. The main objective of this study was to estimate the prevalence of SCD; the secondary objective was to evaluate its association with malaria and respiratory infections.

**Methods:**

This is a cross-sectional study which was carried out in a rural village in the south east coast of Madagascar between May 2011 and November 2013. Participants were children aged between 2–59 months presenting with fever measured by axillary temperature ≥37.5 °C at inclusion. Genotyping of haemoglobin S was done by PCR and malaria was diagnosed by Rapid Diagnostic Test. Research for viral and atypical bacterial respiratory pathogens was performed on nasopharyngeal swabs. Uni-and multivariate polytomous logistic regression was done to assess associations between microbiological results and SCD status, with HbAA phenotype as reference.

**Results:**

A total of 807 children were analysed. Prevalence of SCD among febrile children was 2.4% (95% CI, 1.5–3.7%) and that of SCT was 23.8% (95% CI, 20.9–26.9%). There was no difference in the prevalence of malaria infection according to haemoglobin status (*p* = 0.3). Rhinovirus (22.5%), adenovirus (14.1%), and bocavirus (11.6%) were the most common respiratory pathogens detected. After univariate analysis, patients with SCD were more frequently infected by parechovirus (*p* = 0.01), while patients with SCT were more prone to RSV A or B infection (*p* = 0.01). After multivariate analysis, HbAS phenotype was associated with higher risk of RSV A and B infection compared to HbAA (adjusted *OR* = 1.9; 95% CI: 1.2–3.1, *p* = 0.009), while HbSS phenotype was associated with higher risk of parechovirus infection (adjusted *OR* = 6.0; 95% CI: 1.1–31.3, *p* = 0.03) compared to HbAA, independently of age, gender, period per quarter, and the other viruses.

**Conclusion:**

The prevalence of SCD among under-five children presenting with fever was high in the study population. No association was found between SCT and malaria but few viruses, especially parechovirus, seem to play an important role in the occurrence of pneumoniae among SCD patients.

## Background

During its 59th World Health Assembly held in 2006, WHO recognized sickle-cell disease (SCD), an inherited disorder of haemoglobin as a priority of public health. A resolution was adopted to develop and strengthen efforts for its prevention and management. Sickle-cell anaemia, one of the most common forms of SCD, is due to a point mutation within the sixth codon of the β-globin chain. The produced abnormal variant – haemoglobin S (HbS) – is responsible for chronic haemolytic anaemia and vaso-occlusion which are the underlying causes of the clinical presentation of SCD. Individuals who express the homozygous form (HbSS) manifest the disease, while those with the heterozygous form (HbAS), also known as sickle-cell trait (SCT), are usually asymptomatic carriers.

Initially limited to the sub-Saharan Africa, the Middle-East and some parts of India [1], SCD has currently spread to all continents with the migration of populations. The βS-globin gene is found on five common haplotypes in Africa (Bantu, Benin, Cameroon and Senegal) and Asia (Saudi Arab-Indian) [2] that can be used as a marker of genetic diversity and population origin. Approximately 300,000 children with severe haemoglobin disorders are born every year worldwide [3] and over 75% of SCD occur in Africa where carrier frequency ranged from 1% (Central African Republic, Senegal) to 38% (United Republic of Tanzania) [1]. In Madagascar, several studies conducted in 1950s had estimated the general prevalence of SCD from 4% in the Central highlands [4] to 11-13% in the South-eastern coast [5]. Significant ethnical variation and high prevalence in asymptomatic carrier up to 28% were also reported [6]. To our knowledge, the last published study was done in the early 1980s [7]. These studies, however, were based on the detection of sickle red blood cells in hypoxic conditions, also known as “sickling test”, and the sickle solubility test [3] now considered as obsolete techniques.

In area where malaria is endemic, its relation with SCT was often studied, in particular the high degree of resistance to severe and complicated malaria in sickle cell trait [8, 9]. Malaria is endemic in Madagascar with strong and persistent transmission in the east coast [10].

From May 2011 to November 2013, a cross-sectional study was conducted in the rural village of Ampasimanjeva in the south east cost of Madagascar aiming to identify blood-borne protein biomarkers that can differentiate the causes of unexplained acute febrile illness in children. The study site is known for its high endemicity to malaria, acute respiratory infections and SCD. The main objective of this study was to estimate the prevalence of SCD; the secondary objective was to evaluate its association with malaria and respiratory infections.

## Methods

### Study design and population

This is an observational cross-sectional study with prospective data collection of children aged between 2 to 59 months presenting with fever (axillary temperature ≥37.5 °C according to WHO criteria). The study was conducted from May 2011 to November 2013 in the rural village of Ampasimanjeva located in the south east cost of Madagascar. Ampasimanjeva is 320 km away from the capital city Antananarivo with a total inhabitant estimated to be 22,000 according to the last census. The east coast has hot and humid subequatorial climate and an annual high cumulative rainfall of 4,000 mm per year. Transmission of malaria in this region is reported to be strong and intense all over time. A standardized questionnaire on socio-demographic and clinical data was fulfilled for each individual. The Ampasimanjeva community hospital is a Hospital District Centre receiving each year around 950 children between 2 and 5 years old. Acute Respiratory Infections were the main cause of morbidity accounting for 35% of consultation all ages combined.

Ethical approval for the study was obtained from the National Ethical Committee of the Ministry of Health of Madagascar (authorization 019-CE/MINSAN as of 09/04/2010). Individual written informed consent was provided by the parents of all study participants.

### Sample collection

For each child, venous blood was collected with safety blood containers on dry and spray-dried EDTA. The samples were transported in a refrigerated container at +4 °C to the Centre d’Infectiologie Charles Mérieux for analysis. Transport of samples was organized twice a week.

### Genotyping of haemoglobin

After DNA extraction (QIAamp DNA Mini Kit, Qiagen, Germany) of the EDTA blood samples, the mutation responsible for the generation of HbS was identified by Restriction Fragment Length Polymorphism (RFLP) assay of PCR-amplified DNA (Bio-Rad, USA) [11]. The RFLP pattern of the normal profile (HbAA) of healthy subject is characterized by the presence of the restriction endonuclease Dde*I* site and cleaved in 2 fragments (189 and 93 bp). The homozygous profile (HbSS) responsible of the disease has no restriction site and only one fragment of 282 bp is observed. The heterozygous profile (HbAS) also known as sickle-cell trait combines restriction and no restriction site and reveals 3 fragments (93, 189, and 282 bp).

### Malaria assays

Rapid Diagnostic Test (RDT) of malaria was performed on site using CareStart™ Malaria (Access Bio, Inc., New Jersey, USA) and parasite density was estimated microscopically from the positive samples as previously described [12].

### Detection of respiratory pathogens

Bacterial and viral DNAs as well as viral RNA were extracted from the nasopharyngeal swabs using RTP® Pathogen kit (STRATEC Molecular, Germany). A multiplex real-time PCR assay allowing the identification of 22 respiratory pathogens (Fast-track Diagnostics respiratory pathogens kit, FTD Luxembourg) was used as previously described [13]. Following nucleic extraction of EDTA-whole blood samples, another multiplex real-time PCR assay was performed to identify *Staphylococcus aureus*, *Streptococcus pneumoniae* and *Haemophilus influenzae* type b as previously described [14]. All RNA/DNA amplifications and detections were done using Bio-Rad CFX96 machine (Bio-Rad, USA).

### Statistical analysis

Categorical variables were described as number and percentage with their 95% confidence intervals (CI); continuous covariates were described as median and interquartile range (IQR). The Chi2 test or Fisher’s exact test was used to compare categorical variables. Continuous covariates were compared by Mann–Whitney *U*-test or Kruskal–Wallis one-way analysis of variance. Univariate and multivariate polytomous logistic regression analysis was done to assess microbiological results associated with haemoglobin status, where the normal phenotype was the reference (HbAA).

Statistical analyses were done using Epi Info™ 7.1.3 (Centers for Disease Control and Prevention, Atlanta, Georgia) and Stat 13.0 (StataCorp). All tests were 2-tailed and a *p*-value of less than 0.05 was considered significant.

## Results

### Sample size of eligible children

There were 862 children enrolled in the survey. Among them, 55 were excluded because not meeting the age and temperature criteria and because of inadequate blood sample. Subsequently, a total of 807 children were selected for the final analysis.

### Demographic and health status

Out of the 807 children participating in the survey, 401 (49.7%) were male and the median age was 20 months (IQR: 10–36 months). The median temperature at inclusion was 38.8 °C (IQR: 38.5–39.4 °C). To assess the weight gain and growth, anthropometric parameters of children were measured [15]. The median upper arm circumference was 140 mm (IQR: 132–146 mm). The median weight-for-height Z-score was−1.5 (IQR:−2,2; 0.7); and 245 (30.5%) had weight-for-height Z-score ≤−2SD. The main reasons for medical consultation were malaria (36.6%), pneumonia (33.2%), acute respiratory infection (28.5%), and asthma (1.6%). Pneumococcal Conjugate Vaccine was introduced in 2012 and the national coverage was 76% in 2013 according to WHO and UNICEF estimates. However no information on the vaccination status against *S. pneumoniae* is available among the study population.

### Genotyping of haemoglobin S

To identify the genotype of haemoglobin, the PCR-products from all 807 blood samples were digested and analyzed by the RFLP method. The results of the first 100 genotyping assay were compared to those of the electrophoresis of haemoglobin on alkaline (pH 8.5) agarose gels (Hydragel 7 Hemoglobine, Sebia, Evry, France) and 100% concordant identification was observed (data not shown). Overall, 73.9% (95% CI, 70.7–76.8%) children expressed the normal phenotype HbAA, whereas the prevalence of SCT (heterozygous HbAS) was 23.8% (95% CI, 20.9–26.9%) and that of SCD (homozygous HbSS) was 2.4% (95% CI, 1.5–3.6%).

To classify the nutritional status of the study population, we evaluated the mean weight-for-height Z-score. Individuals with HbSS showed a lower value [−2.2 SD (−3.4;−1.2)] compared to those with HbAS [−1.2 SD (−2.2;−0.5)] or HbAA [−1.5 SD (−2.2; −0.7)] phenotypes (*p* = 0.02), suggesting a state of undernutrition (Table 1). These findings were supported by a significant difference in the values of mid-upper arm circumference between the 3 groups (*p* = 0.001). We also found a higher prevalence of males among individuals with HbSS (68.4%) compared to the HbAS (42.7%) and HbAA (51.3%) groups (*p* = 0.03).

**Table 1:** Characteristics of study population by haemoglobin phenotype

Characteristics	HbAA (*n* = 596)	HbAS (*n* = 192)	HbSS (*n* = 19)	*p*
Period, quarter, N (%)				
- January-March	72 (12.1)	31 (16.1)	3 (15.8)	0.19
- April-June	195 (32.7)	57 (29.7)	7 (36.8)	
- July-September	135 (22.6)	54 (28.1)	6 (31.6)	
- October-December	194 (32.5)	50 (26.0)	3 (15.8)	
Age, months, median (IQR)	21.0 (10.5–36.0)	20.0 (9.0–36.5)	17.0 (10.0–33.0)	0.50
Age category, months, n (%)				
- 2–11	160 (26.8)	62 (32.3)	6 (31.6)	0.68
- 12–23	152 (25.5)	45 (23.4)	5 (26.3)	
- 24–59	284 (47.7)	85 (44.3)	8 (42.1)	
Gender, n (%)				
- Male	306 (51.3)	82 (42.7)	13 (68.4)	0.03
- Female	290 (48.7)	110 (57.3)	6 (31.6)	
Temperature, °C, median (IQR)	38.8 (38.6–39.4)	38.8 (38.6–39.4)	38.5 (38.0–38.7)	0.11
Height, cm, median (IQR)	78.0 (71.0–87.0)	77.0 (69.5–86.0)	74.0 (69.0–86.0)	0.18
Weight, kg, median (IQR)	9.5 (8.1–11.6)	9.3 (7.9–11.4)	8.5 (7.2–10.0)	0.61
Mid-upper arm circumference, cm, median (IQR)	140 (133–146)	140 (132–148)	128 (117–136)	0.001
Weight-for-height Z-score ≤2 SD	−1.5 (−2.2; −0.7)	−1.2 (−2.2; −0.5)	−2.2 (−3.4; −1.2)	0.02
Positive RDT malaria, n (%)	227 (38.1)	63 (32.8)	5 (26.3)	0.3
Naso-pharyngeal carriers positive detection, n (%)				
- Parechovirus	9 (1.6)	1 (0.6)	2 (11.1)	0.02
- RSV A and B	56 (9.8)	30 (17.0)	0 (0)	0.01
- Human coronavirus 229E	6 (1.1)	0 (0)	1 (5.6)	0.07
- Viral coinfection	144 (25.3)	34 (19.2)	8 (44.4)	0.03
*S. pneumoniae* positive from blood, n (%)	11 (1.9)	3 (1.6)	1 (5.3)	0.5

* HbAA, normal phenotype, *HbAS* sickle cell trait, *HbSS* sickle cell disease

### Malaria RDT and microscopy

**Fig. 1:**
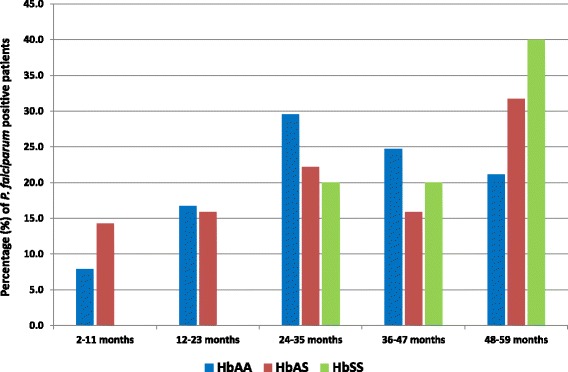
Prevalence of malaria-infected children by age group and haemoglobin status

Out of the 807 children, RDT showed 295 (36.6%; 95% CI, 33.3–39.9%) positive results of which 273 Giemsa-stained thick smear tests were done. The microscopic results showed that 197/273 (72.2%) infections were caused by *Plasmodium falciparum*, 2/273 (0.7%) were due to both *P. falciparum* and *P. vivax*, and the others were negative (74/273, 27.1%). There was no association between haemoglobin status and malaria infection (Table 1). Strikingly, no children with HbS below 2 years of age were infected; however, a trend in an increased detection rate of *P. falciparum* in older children with HbAS and HbSS was observed (Fig. 1).

### Respiratory pathogens infection

Overall, 698 (91.4%; 95% CI, 89.1–93.2%) were positive for at least 1 bacterial pathogen and 575 (75.3%; 95% CI, 72.1–78.3%) for at least 1 viral pathogen. Co-detection of at least two viruses or both bacteria and virus was observed in 186 (24.4%; 95% CI, 21.4–27.6%) and 527 (69.0%; 95% CI, 65.5–72.2%) of cases, respectively. Carriage rate of *S. pneumoniae* was very high (88.9%; 95% CI, 86.4–91.0%) and reached all (100%) children in the HbSS group compared to the HbAS (89.8%; 95% CI, 84.4–93.9%) and HbAA (88.2%; 95% CI, 85.2–90.7%) groups.

**Fig. 2:**
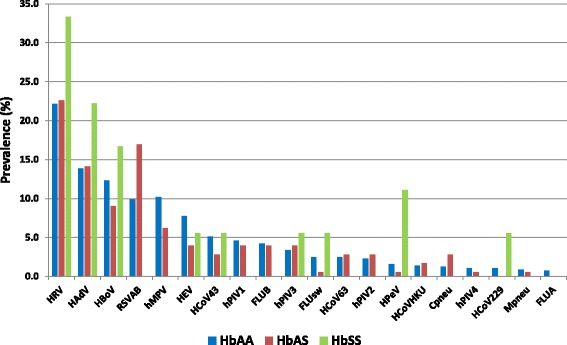
Viral respiratory and atypical bacteria pathogens detected in the nasopharyngeal swabs of study children according to haemoglobin phenotype. HRV: human rhinovirus; HAdV: human adenovirus; HBoV: human bocavirus; RSV: respiratory syncytial virus A and B; hPIV: human parainfluenzavirus 1, 2, 3 or 4; hMPV: human metapneumovirus A or B; HCoV: human coronaviruses NL63, 229E, OC43 or HKU1; HEV: enterovirus; FLU: influenza viruses A, B or A (H1N1)/pdm09; HPeV: human parechovirus; Cpneu: *Chlamydia pneumoniae*; Mpneu: *Mycoplasma pneumoniae*

Out of 764 nasopharyngeal samples tested, rhinovirus (22.5%; 95% CI, 19.7–25.6%; *n* = 172), adenovirus (14.1%; 95% CI, 11.8–16.8%; *n* = 108), bocavirus (11.6%; 95% CI, 9.6–14.1%; *n* = 89), respiratory syncytial virus A or B (11.3%; 95% CI, 9.2–13.7%; *n* = 86), and human metapneumovirus A and B (9.0%; 95% CI, 7.1–11.3%; *n* = 69) were the most common respiratory pathogens detected; *Chlamydia pneumoniae* and *Mycoplasma pneumoniae* accounted for < 2.0% of cases (Fig. 2).

Parechovirus was most frequently detected from respiratory sample in HbSS patients (*n* = 2, 11.1%) compared to the HbAA (*n* = 9, 1.6%) and HbAS (*n* = 1, 0.6%) patients (*p* = 0.02). Similar results were observed with human coronavirus 229E where patients in the HbSS group yielded high positive result (*n* = 1, 5.6%) compared to the HbAA (*n* = 6, 1.1%) and HbAS (*n* = 0) groups (*p* = 0.07) (Table 1). Conversely, respiratory syncytial virus A and B infection were more frequent in HbAS individuals (*n* = 30, 17.0%) compared to HbAA (*n* = 56, 9.8%) and HbSS (*n* = 0) patients (*p* = 0.01). Viral coinfection were frequent in the HbSS group (*n* = 8, 44.4%) compared to the HbAA (*n* = 144, 25.3%) and HbAS (*n* = 34, 19.2%) groups (*p* = 0.03).

Univariate polytomous regression disclosed the risk of respiratory syncytial virus A and B which was 2-fold more higher in HbAS compared to HbAA groups (crude odds ratio [OR] = 1.9; 95% CI: 1.2–3.0, *p* = 0.01), and the risk of parechovirus infection which was higher in HbSS compared to HbAA groups (crude *OR* = 7.8; 95% CI: 1.6–38.9; *p* = 0.01) [Table 2]. After multivariate analysis, HbAS compared with HbAA phenotype remained associated with higher risk of RSV A and B infection (adjusted *OR* = 1.9; 95% CI: 1.2–3.1, *p* = 0.01), independently of age, gender, period per quarter, and parechovirus infection. HbSS compared with HbAA phenotype remained associated with higher risk of parechovirus infection (adjusted *OR* = 6.0; 95% CI: 1.1–31.3.1, *p* = 0.03), independently of age, gender, period per quarter, and RSV A and B infection.

**Table 2:** Microbiological findings associated with heamoglobin status, univariate polytomous logistic regression

Microorganism	Heterozygous form HbAS (*n* = 177)		Homozygous form HbSS (*n* = 19)	
	Crude odds ratio (95% CI)*	*p*	Crude odds ratio (95% CI)*	*p*
> = 1 bacteria from blood	0.8 (0.4–2.0)	0.7	3.8 (1.04–14.2)	0.04
> = 1 bacteria from respiratory sample	1.2 (0.6–2.2)	0.62	NE	–
> = 1 virus from respiratory sample	0.9 (0.6–1.3)	0.57	0.5 (0.2–1.3)	0.15
Coinfection from respiratory sample	0.9 (0.6–1.3)	0.65	0.7 (0.3–1.8)	0.44
Viral coinfection from respiratory sample	0.7 (0.5–1.1)	0.1	2.4 (0.9–6.1)	0.08
Blood				
*S. pneumoniae*	0.8 (0.2–3.1)	0.8	3.0 (0.4–24.1)	0.31
*S. aureus*,	0.8 (0.2–3.1)	0.8	3.0 (0.4–24.1)	0.31
*H. influenzae*	0.4 (0.05–3.6)	0.44	4.7 (0.5–40.0)	0.16
Respiratory sample				
*S. pneumoniae*	1.2 (0.7–2.0)	0.56	NE	–
*S. aureus*	1.6 (0.9–2.9)	0.14	1.9 (0.4–8.4)	0.42
*H. influenzae*	1.0 (0.6–1.7)	0.85	0.4 (0.05–3.0)	0.37
*Mycoplasma pneumoniae*	0.6 (0.07–5.5)	0.68	NE	−
*Chlamydia pneumoniae*	2.3 (0.7–7.4)	0.15	NE	−
Respiratory syncytial virus A and B	1.9 (1.2–3.0)	0.01	NE	−
Human metapneumovirus A and B	0.6 (0.3–1.1)	0.11	NE	−
Human rhinovirus	1.0 (0.7–1.5)	0.9	1.8 (0.6–4.8)	0.27
Human parainfluenzavirus 1	0.9 (0.4–2.0)	0.73	NE	−
Human parainfluenzavirus 2	1.2 (0.4–3.5)	0.68	NE	−
Human parainfluenzavirus 3	1.2 (0.5–2.9)	0.7	1.7 (0.2–13.5)	0.6
Human parainfluenzavirus 4	0.5 (0.06–4.5)	0.56	NE	−
Human coronavirus 229E	NE	−	5.5 (0.6–48.4)	0.12
Human coronavirus NL63	1.2 (0.4–3.2)	0.79	NE	−
Human coronavirus OC43	0.5 (0.2–1.4)	0.21	1.1 (0.1–8.5)	0.93
Human coronavirus HKU1	1.2 (0.3–4.6)	0.78	NE	−
Influenza virus A	NE	−	NE	−
Influenza virus A (H1N1)/pdm09	0.2 (0.03–1.7)	0.15	2.3 (0.3–18.8)	0.43
Influenza virus B	0.9 (0.4–2.2)	0.88	NE	−
Human parechovirus	0.4 (0.04–2.8)	0.32	7.8 (1.6–38.9)	0.01
Human adenovirus	1.0 (0.6 (1.7)	0.94	1.8 (0.6–5.5)	0.32
Human bocavirus	0.7 (0.4–1.3)	0.24	1.4 (0.4–5.0)	0.58
Enterovirus	0.5 (0.2–1.1)	0.09	0.7 (0.09–5.4)	0.73

*After polytomous univariate logistic regression, compared with normal form HbAA (*n* = 569)

NE, non estimable

Out of the 807 whole blood samples tested, invasive pneumococcal disease was reported in 15 patients (1.9%; 95% CI, 1.1–3.3%). All of them recovered well without complication.

## Discussion

Our study shows that prevalence of SCD was 2.4% and that of SCT 23.8% among children aged 2–59 months presenting with fever and living in high endemic area for malaria. To our knowledge, this study is the first to document the prevalence of HbSS in febrile young children. Although no similar age group is available for comparison, previous results report high carrier rate of 29.6% to 31.9% [16, 17] from the same ethnic group in which the βS-globin gene frequency was reported to be 0.159 [17]. We cannot certify however the ethnicity of our studied population because it has not been queried during the investigation. In an extensive study of 1,214 individuals of 14 different ethnic groups aged 2–6 years conducted in 2007 (Institut Pasteur de Madagascar, unpublished data), a much lower prevalence of 0.2% for HbSS and 9.4% was found for the heterozygous state compared to our study. When data from the South-east coast only is selected in this later study, the prevalence of HbSS is 0.7% and that of the heterozygous state is 18.7%, slightly lower than that observed in our study. While these data show a higher SCT prevalence among the study population in Madagascar, further study is needed to estimate the real burden among the general population.

Our results demonstrate a significantly lower mean weight-for-height Z-score for patients with SCD than those of normal children and individuals with SCT. Previous studies from other developing countries have reported a state of undernutrition among patients with SCD [18, 19] that may lead to slow growth and maturational abnormality, amid other complications [20]. These results confirm previous observations that nutritional supplementation is a major component in the management of SCD.

Studies reporting gender differences in SCD incidence are rare and mainly focus on the occurrence of crises or comorbidities [21, 22]. However, in a cross-sectional study carried out over 735 students in India, the prevalence of SCT using the sickling test was higher among male (7.3%) compared to that among female (5.9%) [23]. Our study found higher SCD prevalence among male population, but no significant difference between male and female for SCT prevalence. This should be taken into account in the gender-selective use of medical facilities and management of patients.

Previous studies have shown that SCT protects against severe forms [8] and mild malaria infections [24, 25], although the precise mechanism remains poorly understood. In our study, a high proportion of *P. falciparum* positive result (36.6%) has been found among the studied population. Overall, we did not find any statistical difference between malaria-infected children in the HbAS group compared to the normal phenotype. However, as the age increases, the frequencies of children in the HbAS and HbSS groups who experienced malaria increases. The absence of malaria infection in the patients with SCD up to 2 years of age may have several causes including an early death due to malaria or other infections.

Individuals with SCD are strongly susceptible to encapsulated bacterial organisms such as *S. pneumoniae*, a common colonizer of the nasopharynx in healthy children. Here we report a very high pneumococcal carriage rate among the febrile children studied. Strikingly, all children (100%) with homozygous sickle cell disease in our study were carriers. In previous reports, a much lower rate was found in the US (12%) [26] and Uganda (33%) [27] during study conducted on patients with homozygous sickle cell disease. In our study, pneumonia and acute respiratory infections were the main reasons for medical consultation after malaria. However, the role of *S. pneumoniae* in the development of the disease in the majority of patients is hypothetical due to the absence of healthy matched control group. It should be mentioned that 1.9% of the patients had bacteraemia with *S. pneumoniae* during the course of infection but its causative role has not been proven up as up to 5% of febrile children may also develop asymptomatic or occult bacteraemia [28].

The overall prevalence of viral infection/detection among the study population was high independent of haemoglobin status. However, among the 23 viral and atypical bacteria pathogens studied, there was no statistical difference in prevalence between the three groups for 20 of them and include HRV the most common respiratory virus detected or influenza virus A (H1N1)/pdm09 known to increase disease severity in children with SCD [29]. Parechovirus, a member of the family *Picornaviridae*, was significantly associated with a high risk of infection in SCD patients in our study. Parechovirus infections are usually asymptomatic or lead to a mild gastro-intestinal or respiratory diseases. Only two patients had parechovirus detected, both presenting with symptoms of pneumoniae, suggesting that larger prospective study is required to better describe the real impact of parechovirus infection among SCD population. Conversely, RSV A or B belonging to the family *Paramyxoviridae*, was significantly associated with a high risk of infection in SCT patients compared to the normal population whereas no virus was detected in SCD patients. RSV A and B are a major cause of lower respiratory tract infection during infancy and childhood and were associated with acute chest syndrome similar in severity to influenza infection in febrile children with SCD [30]. The reason why RSV A and B were not detected in our study using a sensitive molecular tool is unknown but it may be linked either to the small number of identified SCD patients, or the possible death of those children. However no follow-up of the study population was carried out to ascertain the latter hypothesis.

Detection of respiratory viruses has been facilitated by the recent widespread availability of nucleic acid amplification techniques. In a study carried out previously in Ampasimanjeva to describe the aetiology of a clinically defined cohort of children with fever and acute respiratory infections, the most representative pathogens were human metapneumovirus (hMPV) for pneumonia, human parainfluenzavirus (hPIV) for other acute lower respiratory infections, respiratory syncytial virus (RSV) for flu-like illnesses and human adenovirus (HAdV) for upper respiratory tract infections [13]. However, the clinical significance of viral detection in biological samples from patients with respiratory infections remains often unclear. For example, when detected in a patient with severe pneumonia, respiratory viruses may represent subclinical infection, persistent shedding after a prior infection, infection restricted to the upper respiratory tract, or infection involving the lower respiratory tract [31–33]. While some pathogens, such as RSV, hMPV and FLU seem to be strict pathogens, other viruses such as HRV are widely identified in both cases and controls. It was beyond the scope of this study to evaluate the viral distribution in asymptomatic patients to study proportion of viral shedding in a control group, or to distinguish between upper and lower respiratory tract infections. Nevertheless, the same study conducted a year ago in the same population has confirmed our findings that a wide range of respiratory viruses might have played a role in escalating respiratory tract viral infections among patients with SCD and SCT.

Our study was limited by the fact that the genotyping method used may not be totally specific to detect haemoglobin S as the presence or the absence of the restriction site Dde*I* may be found in other hemoglobinopathies. However, according to one recent publication [34], HbS seemed to be the most prevalent in Africa where over 700 haemoglobin variants were identified. Additionally, the association between sickle cell trait and malaria is not conclusive for the following reasons: the initial design was probably not optimal to detect this association in the current study, and more accurate methods for detecting HbS should have been used (i.e. hemoglobin electrophoresis). Further independent and well-structured research should be considered in that matter. A second limitation of this study is the absence of long-term follow-up to estimate the outcomes of children and the proportion and causes of death mainly among those living with SCD.

## Conclusion

Our findings show that the prevalence of SCD among under-five children presenting with fever was high and a better knowledge of the role and distribution of viral and atypical bacteria pathogens in the study population has been found. This study has helped moving towards a better understanding of the epidemiology of SCD, and we hope it will trigger further research in that direction, for the following reasons. Firstly, as SCD is a major public health concern in Madagascar, a large prospective study on the general population to estimate the true magnitude of the disease should be done. Secondly, it is important to document data about the clinical course and mortality associated to SCD in regions where it is endemic. Thirdly, study involving increasing laboratory capacity should be conducted to help a better understanding of SCD epidemiology, as well as other hemoglobinopathies. Finally, conclusions from these studies will be used for awareness-raising among national authorities in order to strengthen the national control program.

## Abbreviations

SCD: Sickle-cell disease
SCT: Sickle-cell trait
HbSS: Homozygous sickle haemoglobin
HbAS: Heterozygous sickle haemoglobin
HbAA: Normal haemoglobin
RFLP: Restriction Fragment Length Polymorphism assay
RDT: Rapid Diagnostic Test
